# Genetic Alteration Profiling of Chinese Lung Adenocarcinoma and Its Effect on Targeted Therapy Efficacy

**DOI:** 10.3389/fonc.2021.726547

**Published:** 2021-12-14

**Authors:** Jie Liu, Wang-yang Xu, Maosong Ye, Zilong Liu, Chun Li

**Affiliations:** ^1^ Department of Pulmonary and Critical Care Medicine, Zhongshan Hospital, Fudan University, Shanghai, China; ^2^ Department of Medicine, Singlera Genomics (Shanghai) Ltd., Shanghai, China

**Keywords:** lung adenocarcinoma, next-generation sequencing (NGS), genetic alteration, tumor mutation burden (TMB), targeted therapy

## Abstract

**Background:**

Non-small cell lung cancer (NSCLC) is the most common type of lung cancer and a highly heterogeneous disease with a diversity of phenotypes and genotypes in different populations. The purpose of this study is to investigate oncogenic alterations of lung adenocarcinoma (LUAD) in eastern China and their significance in targeted therapies.

**Methods:**

This study enrolled 101 LUAD patients and used a customized DNA panel to detect molecular alterations. Comprehensive analysis of mutations and clinical application of genomic profiling was carried out.

**Results:**

The most commonly mutated genes were epidermal growth factor receptor (*EGFR*) (53%) and tumor protein p53 (*TP53*) (32%). The less frequently mutated genes were erb-b2 receptor tyrosine kinase 2 (*ERBB2*) (25%), ATR serine/threonine kinase (*ATR*) (20%), CCAAT enhancer binding protein alpha (*CEBPA*) (16%), RB transcriptional corepressor 1 (*RB1*) (16%), transcription factor 7 like 2 (*TCF7L2*) (14%), ROS proto-oncogene 1, receptor tyrosine kinase (*ROS1*) (12%) and spectrin alpha, erythrocytic 1 (*SPTA1*) (12%). Among them, the frequency of *ERBB2*, *ATR*, *CEBPA*, *RB1* and *TCF7L2* mutations was much higher than that in the databases. Seventy percent of the patients harbored at least one actionable alteration according to the OncoKB evidence. *CEBPA* mutations affected the efficacy of *EGFR*-tyrosine kinase inhibitors. *ERBB2*, *CEBPA* and *TCF7L2* mutated tumors tend to have higher tumor mutation burden (TMB).

**Conclusions:**

LUAD patients from eastern China have a unique profile of mutations. The targeted DNA panel is helpful for personalized treatment decision of LUAD patients, and specific mutations may affect the efficacy of targeted therapies.

## Introduction

Lung cancer is the most common cancer in China (an incidence rate of 35.10/100000, 774323 new cases) (1). NSCLC accounts for the most (80–85%) of all lung cancers and is mainly divided into LUAD, squamous cell carcinoma (SCC) and large cell carcinoma (LCC) (2). Targeted therapies have greatly prolonged the survival time of NSCLC patients harboring actionable driver alterations (3, 4). Besides, the application of immune checkpoint inhibitors (ICIs), has also significantly improved the overall survival (OS) of some lung cancers (5, 6). Over the last decade, next-generation sequencing (NGS) testing is increasingly used for clinical diagnosis and therapies (7–11). An NGS cancer gene panel (CGP) was applied to detect actionable driver alterations and measure TMB. Understanding the characteristics of molecular alterations in lung cancer can help patients choose personalized targeted therapy or immunotherapy.

Population reports focusing on Europe, America, South Korea, and China showed differences in the frequency of driver genes among lung cancer patients, which affect the efficacy of targeted drugs (12–15). Thus, it is necessary to study the genomic profiles of LUAD in Chinese patients. A total of 101 LUAD samples were analyzed by a targeted panel of 639 genes (Singlera OncoAims® Panoramic Detection Panel). This panel refers to cancer databases, clinical guidelines and references to detect genetic alterations including targeted drug related actionable alterations and uncommon mutations that have not been well studied in LUAD. Second, we demonstrated the utility of DNA sequencing results to guide clinical treatment and explored the impact of mutations on the efficacy of *EGFR*-targeted therapy to identify the predictive biomarkers.

## Materials and Methods

### Patients and Samples Collection

We enrolled 101 patients with diagnosed LUAD in Zhongshan Hospital Affiliated to Fudan University from January 2018 to March 2021. Two pathologists determined the histological type and degree of differentiation according to the 2015 WHO classification of lung tumors (16), and the disease staging according to the Union for International Cancer Control (UICC) classification of the tumor-node-metastasis (TNM) (17). Patients with other malignancies and multiple malignancies were not included in this study. The basic clinical information included age, gender, histological diagnosis, TNM stage, degree of differentiation, metastasis, and smoking history. Patients were divided into 3 groups according to the smoking status: never-smokers, ever-smokers (ceased >1 year) and current smokers (ongoing or ceased ≤ 1 year). This study was approved by the Ethics Committee of Zhongshan Hospital Affiliated to Fudan University. All participants signed informed consent. Formalin fixed paraffin embedded (FFPE) tumor tissues and paired blood samples were collected.

### Genomic DNA Preparation

According to the manufacturer’s instructions, FFPE DNA was isolated by using the QIAamp DNA FFPE Tissue Kit (Qiagen, Hilden, Germany), and gDNA from blood was extracted by using the QIAamp DNA Blood Mini Kit. The quality of DNA was detected by 1% agarose gel electrophoresis. The Qubit dsDNA HS Assay kit and Qubit 3.0 fluorimeter (Life Technologies, Eugene, Oregon, USA) were used to quantify DNA.

### NGS Analysis and Somatic Alteration Detection

Singlera OncoAim® Panoramic Detection Panel (Singlera Genomics (Shanghai) Ltd., China) was conducted for mutation analysis. According to the Illumina standard library construction instructions (Illumina, Inc., California, USA), 20 ng DNA was prepared to generate libraries by using a KAPA Library Quantification Kit (Kapa, KK4824). The library products were sequenced using 75 bp paired-end runs on the Illumina MiSeq. The sequencing data was processed by bioinformatics software provided by the manufacturer. The minimum sequencing depth and allele frequency (MAF) of single nucleotide polymorphisms (SNPs) was set to ≥200x and ≥ 5%, respectively. Raw data containing sequence information and quality information was aligned to the reference human genome (UCSC hg19). Single nucleotide variants (SNVs), small insertions and deletions (InDels), mixed variants, structural variants and amino acid changes were identified using SnpEff3.0 which is the genetic variant annotation and functional effects prediction tool. SnpEff can annotate and predict the effects of variants on genes and amino acids programmatically (18, 19). Germline and somatic alterations were all detected. Variations exist in tumors but not in matched blood are viewed as somatic mutations. The list of 639 genes was shown in **Supplementary Table 1**.

### Actionable Alterations Analysis

OncokB (Precision Oncology Knowledge Base) website is developed and maintained by the Memorial Sloan Kettering Cancer Center (20). It contains the information of 125 cancer types related 682 genes, 5670 alterations and corresponding 103 drugs. The effects of targeted inhibitors vary with tumor lineages, even in cancers with the same mutant. Potentially actionable changes in specific cancer types are divided into four levels. When the tumor type of non-small cell lung cancer was selected, the “actionable genes” showed the genes and mutant sites/alleles targeted by evidence-based drugs. At present, a total of 8 genes have recommended drugs. When selecting the tumor type of all solid tumor, 10 additional genes have recommended drugs, and most of these mutations are level 4 alterations. These 18 genes are included in the DNA panel.

### Statistical Analysis

Mutational profiling was conducted by using MAF Visualization tools (maftools) in R (version 3.6.1) (http://www.r-project.org) (21, 22). 3-D structures of protein were performed by using I-TASSER server (http://zhanglab.ccmb.med.umich.edu/I-TASSER) (23). Fisher exact test was used to test for significance between two groups. *P*-value < 0.05 was considered statistically significant. For all figures: asterisk (*) means *P*-value <0.05, (**) means *P*-value <0.01, (***) means *P*-value <0.001.

## Results

### Patient Characteristics and Testing Flowchart

**Figure 1:**
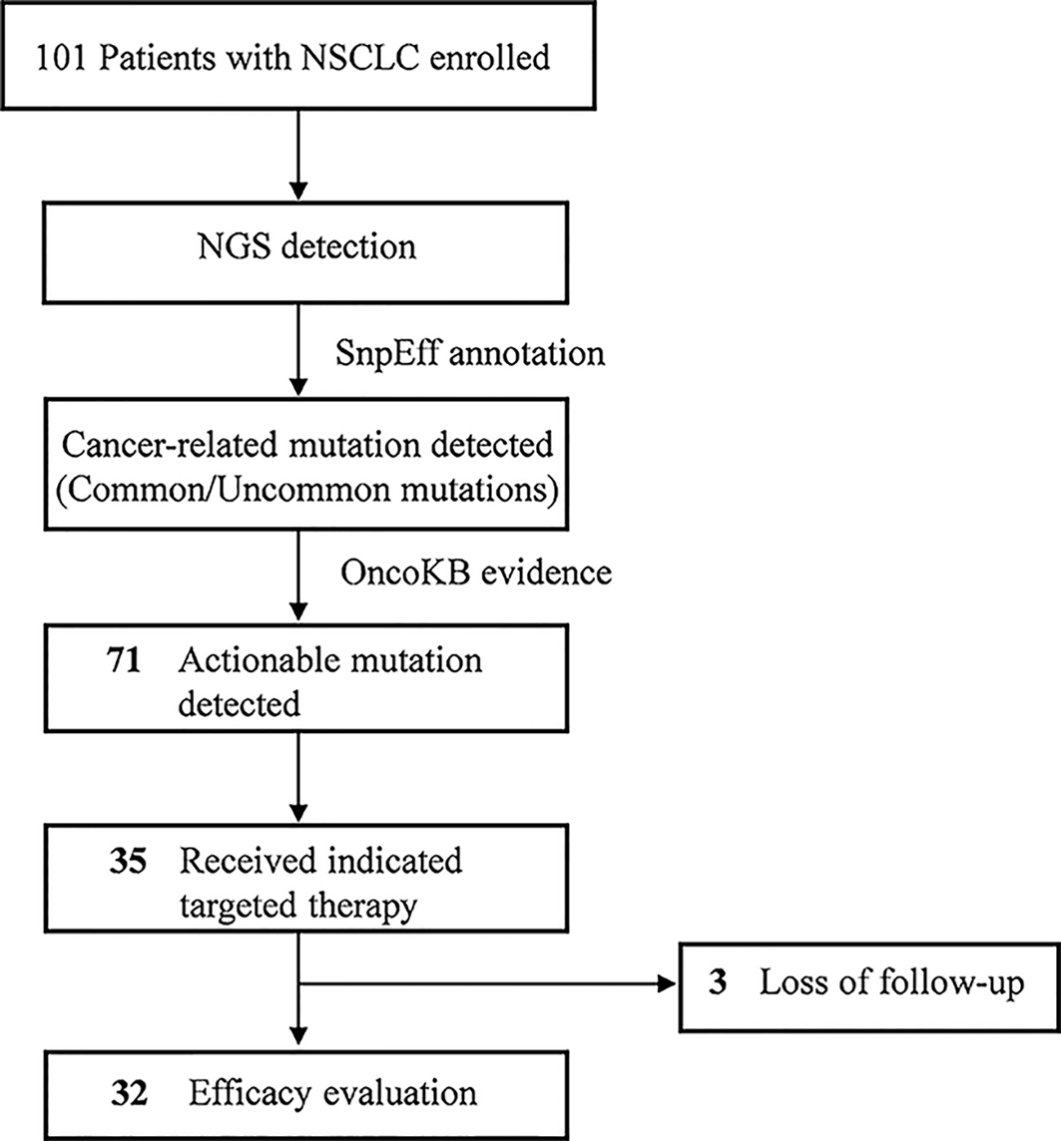
Testing Flowchart of patients. Flowchart indicates patient enrollment, DNA panel conducted, cancer-related mutations detected and targeted therapies available.

In total, 101 patients with LUAD comprising 42 males and 59 females were enrolled in this study and patients were aged 34–88 years, with a median age of 61 years. Overall, 46.5% of the patients were diagnosed with stage I, 16.8% with stage II, 4.0% with stage III, and 32.7% with stage IV LUAD. In total, 4.0% of cases was well differentiated, 55.4% was moderate differentiated, and 40.6% was poor differentiated. Most patients (79.2%) were non-smokers (**Supplementary Table 2**). NGS results were used to analyze common and uncommon mutations. A total of 71 patients had actionable mutations detected in tissues, of which 35 received targeted therapies. There were 32 patients with follow-up information who were evaluated for the efficacy of targeted drugs (**Figure 1**).

### Genomic Landscape of LUAD Patients

**Figure 2:**
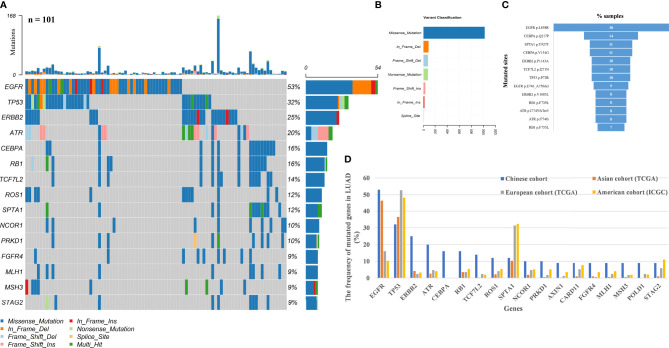
Mutational landscape of 101 Chinese LUAD patients. **(A)** 15 top frequently mutated genes are shown. The X-axis represents the sample of each patient, and the Y-axis represents the mutated genes and the mutation frequency of each mutated gene. **(B)** Display of variation classification. **(C)** High frequency hot spot mutated sites in LUAD patients. **(D)** Comparison of the frequency of 18 significantly mutated genes identified in Chinese LUAD patients with that in the TCGA and ICGC cohorts. TCGA, The Cancer Genome Atlas. ICGC, International Cancer Genome Consortium.

A total of 314 mutated genes were detected by using the DNA panel in LUAD patients, with a mean of 8 alterations per sample. The most commonly mutated genes were *EGFR* (53%, 54/101) and *TP53* (32%, 32/101). Other most frequently affected genes were *ERBB2* (25%, 25/101), *ATR* (20%), *CEBPA* (16%), *RB1* (16%), *TCF7L2* (14%), *ROS1* (12%) and *SPTA1* (12%) (**Figure 2A**). Among these mutations, missense mutation was the most common mutation, followed by in frame deletion and frame shift deletion (**Figure 2B**). Besides, 1.98% (2/101) was *EML4-ALK* fusion and 1.98% (2/101) was *KIF5B-RET* fusion. The most common alteration was *EGFR* p. L858R, followed by *CEBPA* p.Q217P. Multiple mutations including *CEBPA* p.Q217P, *SPTA1* p.Y927F, *CEBPA* p.V154G, *ERBB2* p.P1140A, *TCF7L2* p.I271N, *ERBB2* p.V1085L, *RB1* p.F739L, *ATR* p.I774YfsTer5 and *ATR* p.I774fs have never been reported, but the possibility that they are genetic polymorphisms has been ruled out (**Figure 2C**). The mutation frequency of *ERBB2*, *ATR*, *CEBPA*, *RB1*, and *TCF7L2* genes in the panel was significantly higher than that reported in the the Cancer Genome Atlas (TCGA) (Asian and European cohorts) and International Cancer Genome Consortium (ICGC) (American cohort) databases (**Figure 2D**, **Supplementary Table 3**). Algorithm predicted that fibroblast growth factor receptor 1 (*FGFR1*), KRAS proto-oncogene, GTPase (*KRAS*), NRAS proto-oncogene, GTPase (*NRAS*), *EGFR*, *CEBPA* and *RB1* were prominent driver genes. In addition, *ATR* and *TCF7L2* were also the driver genes (**Supplementary Figure 1**).

**Figure 3:**
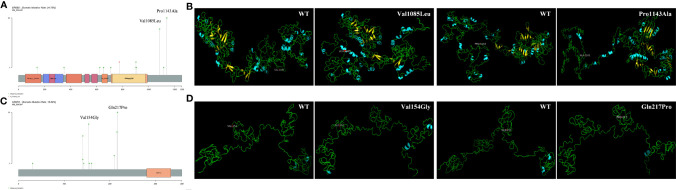
Mutant points and 3-D protein structures of normal and mutant protein of ERBB2 **(A, B)** and CEBPA **(C, D)**. Blue means helix, green means sheet. The mutant points were shown in red in the structure diagram.

The profile of common driver genes in the current Chinese cohort was shown in **Supplementary Figure 2**. We focus on novel carcinogenic mutations found in the sequencing. *ERBB2* (also called *HER2*) is recognized as an oncogene (24, 25). More recently, *ERBB2* gene amplifications and mutations have been reported in 2% to 4% of LUAD (26). In a Chinese study of 98 LUAD patients, the mutation frequency of ERBB2 was 24%, similar to that reported in this Chinese cohort (27). The two sites, *ERBB2* p.P1140A and *ERBB2* p.V1085L with the highest mutation frequency (10% and 8%, respectively) occurred outside the functional domain, but they changed the protein structure, which may affect ERBB2 receptor activation (**Figures 3A, B**). The role of CEBPA was first established in acute myeloid leukemia (AML) (28). *CEBPA* acts as a tumor suppressor and was found to be down regulated in 50% of II and IIIA LUAD (29). But the pathologic mutations of *CEBPA* were uncommon in lung cancer. In this study, missense mutation was the most common variant type, and it could change the structure of protein and contribute to lung malignancies (**Figures 3C, D**). Other genes with relatively high mutation frequency were *ATR*, *RB1* and *TCF7L2* genes. Although they are not the commonly mutated genes in NSCLC, their mutation frequency in this cohort was higher than that in the previously reported cohorts. Among patients, frame shift insertion/deletion of *ATR* and the major mutation of *RB1* (*RB1* p.F739L) and *TCF7L2* (*TCF7L2* p.I271N) changed the protein structure during simulation (**Supplementary Figure 3**), suggesting that they might play an important role in the development of lung cancer. The mutated sites of other 15 mutated genes with high frequency were displayed in **Supplementary Figure 4** (30, 31).

### Targeted Therapy Based on NGS Testing Results

**Figure 4:**
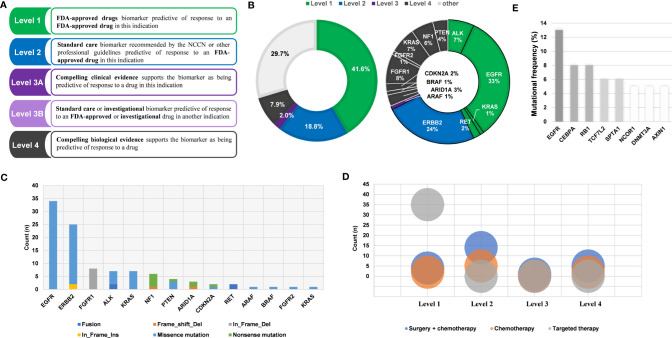
Clinical actionable mutations and their clinical evidence. **(A)** OncoKB levels of evidence. **(B)** Samples were divided into different grades according to mutations (left). Mutated genes in different grades (right). **(C)** Mutation types in actionable alterations. **(D)** Different treatment means in different grades. **(E)** The most frequently mutated genes in patients without actionable alterations.

OncoKB (http://oncokb.org/) evidence was applied to evaluate whether molecular changes can guide treatment (**Figure 4A** and **Table 1**). Of 101 patients, 71 (70.3%) harbored sensitizing mutations and were recommended for targeted therapies according to the OncoKB evidence. Besides, 41.6% of those had matched FDA-recognized level_1 actionable alterations including *ALK* fusion and oncogenic mutations, *EGFR* and *KRAS* mutations and *RET* fusion, 18.8% had level_2 *ERBB2* alterations, and 2% had level_3 *ARAF* alteration. Level_4 alterations accounted for 7.9% including oncogenic mutations in *ARID1A*, *BRAF*, *CDKN2A*, *FGFR1*, *FGFR2*, *KRAS*, *NF1* and *PTEN*. (**Figures 4B, C**). We found that targeted therapy has become the first choice for patients with level_1 alterations (**Figure 4D**). Among the 36 patients being followed up, 32 patients who used matching targeted drugs had a median remission period > 14 months, and patients No. 33, 34, 35 had mutations of the recommended targeted drug, but they received chemotherapy, and the disease progressed after an average of 7 months. Patient NO. 36 had no clinically available mutation, and his disease progressed 5 months after chemotherapy (**Table 2**). In addition, almost 29.7% of the patients had no clinically applicable mutations of NSCLC, among which *CEBPA*, *RB1*, *TCF7L2* were the most common mutated genes (**Figure 4E**), thus providing potential targets for the research and development of new targeted drugs.

**Table 1:** Mutated genes and actionable targeted therapies.

Level^a^	Gene	Alterations	Tumor type	Targeted therapy guidelines	Actionable mutational frequency, n (%)	Actual targeted therapy according to guidelines, n
1	ALK	Fusions, Oncogenic Mutations	Non-Small Cell Lung Cancer	Alectinib, Ceritinib, Crizotinib, Brigatinib, Lorlatinib	7 (6.9)	2
1	BRAF	V600E	Non-Small Cell Lung Cancer	Dabrafenib+Trametinib	0	/
1	EGFR	19 Del, L858R, Exon20 insertion, G719, L861Q, S768I, T790M	Non-Small Cell Lung Cancer	Afatinib, Dacomitinib, Erlotinib, Gefitinib, Osimertinib, Amivantamab, Mobocertinib	34 (33.7)	33
1	KRAS	G12C	Non-Small Cell Lung Cancer	Sotorasib	1 (1.0)	/
1	NTRK1/2/3	Fusions	All Solid Tunors	Entrectinib, Larotrectinib	0	/
1	RET	Fusions	Non-Small Cell Lung Cancer	Pralsetinib, Selpercatinib	2 (2.0)	/
1	ROS1	Fusions	Non-Small Cell Lung Cancer	Crizotinib, Entrectinib	0	/
2	EGFR	A763_Y764insFQEA	Non-Small Cell Lung Cancer	Erlotinib	0	/
2	ERBB2	Oncogenic Mutations	Non-Small Cell Lung Cancer	Ado-Trastuzumab Emtansine, Trastuzumab Deruxtecan	25 (24.8)	/
3	ARAF	Oncogenic Mutations	Non-Small Cell Lung Cancer	Sorafenib	1 (1.0)	/
3	EGFR	Exon19 insertion, Kinase Domain Duplication	Non-Small Cell Lung Cancer	Erlotinib, Gefitinib, Poziotinib, Afatinib	0	/
4	ARID1A	Truncating Mutations	All Solid Tunors	PLX2853, Tazemetostat	3 (3.0)	/
4	BRAF	G464, G469A, G469R, G469V, K601, L597	All Solid Tunors	PLX8394	1 (1.0)	/
4	CDKN2A	Oncogenic Mutations	All Solid Tunors	Palbociclib, Abemaciclib, Ribociclib	1 (1.0)	/
4	EGFR	D761Y, L718V, L747P	Non-Small Cell Lung Cancer	Afatinib, Osimertinib	0	/
4	FGFR1/2/3	Oncogenic Mutations	All Solid Tunors	Erdafitinib, Infigratinib, Debio1347, AZD4547	9 (9.0)	/
4	KRAS	Oncogenic Mutations	All Solid Tunors	Cobimetinib, Trametinib, Binimetinib	7 (7.0)	/
4	NF1	Oncogenic Mutations	All Solid Tunors	Trametinib, Cobimetinib	6 (6.0)	/
4	PTEN	Oncogenic Mutations	All Solid Tunors	AZD8186, GSK2636771	4 (4.0)	/

a OncoKB levels of evidence.

**Table 2:** Treatment follow-up records.

Sample No.	Gender	Age	TNM stage	Actionable alteration	OncoKB level	Treatment	Drug evidence	Time to remission (months)
1	Female	70	T4N3M1a	EGFR L858R	1	Gefitinib	FDA-approved	25
2	Female	59	T2aN2M1b	EGFR 19del	1	Gefitinib	FDA-approved	18
3	Female	57	T1bNxM1	EGFR L858R	1	Gefitinib	FDA-approved	21
4	Male	56	T4N2M0	EGFR 19del	1	Gefitinib	FDA-approved	12
5	Male	76	T4N2M1b	EGFR L858R	1	Erlotinib	FDA-approved	48
6	Male	88	T4N3M0	EGFR L858R	1	Gefitinib	FDA-approved	13
7	Female	66	T3N3M1c	EGFR L858R	1	Gefitinib	FDA-approved	29
8	Male	63	T4N2M1	EGFR 19del	1	Gefitinib	FDA-approved	32
9	Female	78	T4N3M1c	EGFR 19del	1	Gefitinib	FDA-approved	9
10	Male	62	T3N2M1c	EGFR L858R	1	Erlotinib	FDA-approved	7
11	Male	73	T1cN2M1c	EGFR 19del	1	Gefitinib	FDA-approved	35
12	Male	62	T2aN3M1a	EGFR T790M	1	Osimertinib	FDA-approved	17
13	Male	71	T3N3M1c	EGFR L858R	1	Gefitinib	FDA-approved	7
14	Female	71	T2N2M1c	EGFR L858R	1	Pembrolizumab + Gefitinib	FDA-approved	15
15	Male	73	T2bN0M1c	EGFR T790M	1	Osimertinib	FDA-approved	29
16	Female	75	T4N2M1a	EGFR L858R	1	Gefitinib	FDA-approved	12
17	Female	72	T4N2M1c	EGFR 19del	1	Gefitinib	FDA-approved	10
18	Female	49	T2N2M1c	ALK fusion	1	Alectinib	FDA-approved	8
19	Male	68	T3N3M1a	EGFR 19del	1	Osimertinib	FDA-approved	28
20	Female	52	T2N1M1c	EGFR 19del	1	icotinib	NMPA-approved	4
21	Female	47	T1bN2M1c	EGFR T790M	1	Osimertinib	FDA-approved	14
22	Male	72	cT3N2M1a	EGFR 19del	1	Erlotinib	FDA-approved	5
23	Female	44	cT4N3M1c	EGFR L858R	1	Gefitinib	FDA-approved	6
24	Female	58	cT4N3M1c	EGFR L858R	1	HS-10296	NMPA-approved	12
25	Female	73	cT1cN3M1c	EGFR 19del	1	Gefitinib	FDA-approved	5
26	Female	72	cT4N3M1	EGFR 19del	1	Gefitinib	FDA-approved	7
27	Female	65	T1bN2M0	EGFR L858R	1	Gefitinib	FDA-approved	20
28	Female	55	T2aN2M0	EGFR L858R	1	Gefitinib	FDA-approved	24
29	Female	52	T2aN0M0	EGFR 19del	1	Gefitinib	FDA-approved	22
30	Female	50	T2aN0M0	EGFR T790M	1	Osimertinib	FDA-approved	25
31	Female	65	T2aN0M0	EGFR L861Q	1	Afatinib	FDA-approved	18
32	Male	55	T2aN0M0	EGFR G719A	1	Erlotinib	FDA-approved	16
33	Male	62	T3N2M1c	EGFR L858R	1	Pemetrexed + carboplatin	/	6
34	Male	73	T1cN2M1c	EGFR 19del	1	Pemetrexed + carboplatin	/	8
35	Female	41	T1cN3M1c	ERBB2 p.A775_G776insYVMA	2	Pemetrexed + carboplatin	/	7
36	Male	55	T2aN2M0	/	/	Pemetrexed + carboplatin	/	5

FDA, U.S. Food and Drug Administration; NMPA, China National Medical Products Administration.

### Response to Indicated Targeted Therapy

**Figure 5:**
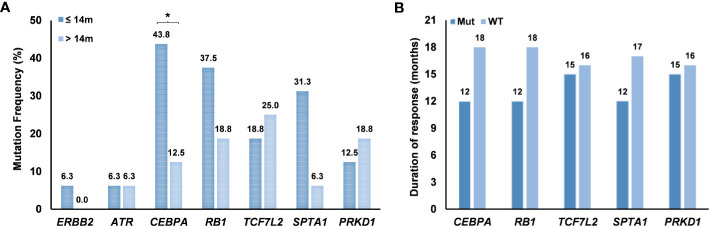
Effect of mutated genes on the efficacy of EGFR-TKIs. **(A)** Differences in mutation frequency of mutated genes between duration of response ≤ 14 months and duration of response > 14 months. **(B)** Duration of response to TKIs according to gene mutations. *P < 0.05. Mut, mutation; WT, wild type.

We investigated the relationship between the driver genes and the duration of EGFR-TKIs response. The results showed that *CEBPA* mutations were more common in patients with short duration of response to TKIs (< 14 months) than that in patients with long duration of response (> 14 months) (43.8% *vs*. 12.5%, *P* =0.031) (**Figure 5A**). The duration of response to TKIs in patients with *CEBPA* mutations was shorter than that in patients without *CEBPA* mutations (12 months *vs*. 18 months) regardless of other driver gene mutations (**Figure 5B**). However, *RB1* and *SPTA1* mutations were common in patients with short duration of response to TKIs. Patients with *RB1* and *SPTA1* mutations had shorter TKI remission time than those without mutations, but there was no significant difference (**Figure 5B**). *TCF7L2* and *PRKD1* mutations were common in patients with longer duration of response, although there was no statistical difference (**Figure 5A**).

### Tumor Mutation Burden of LUAD

**Figure 6:**
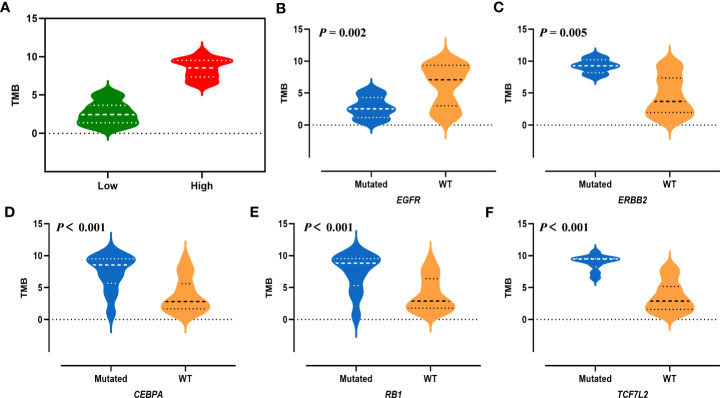
Tumor mutation burden of LUAD. **(A)** The distribution of TMB across all patients, using a threshold of 6.3 mutations/Mb. Low (<6.3 mutations/Mb), and high (≥ 6.3 mutations/Mb). Comparison of TMB according to *EGFR* mutations **(B)**, *ERBB2* mutations **(C)**, *CEBPA* mutations **(D)**, *RB1* mutations **(E)**, *TCF7L2* mutations **(F)**.

TMB is a biomarker of sensitivity to immune checkpoint inhibitors including PD-1 and PD-L1 blockade immunotherapy (32). In the current study, the median TMB of LUAD was 4.82 (range 0.65–10.50) mutations/Mb. According to the TCGA (the Cancer Genome Atlas) database, the average TMB value of lung adenocarcinoma was set as follows: low (<6.3 mutations/Mb) and high (≥ 6.3 mutations/Mb) (**Figure 6A**). *EGFR* mutated tumors had lower median TMB than *EGFR* wild-type tumors (**Figure 6B**). LUAD with somatic mutations in *ERBB2*, *CEBPA*, *RB1* and *TCF7L2* genes got higher TMB than tumors without those mutations (**Figures 6C–F**). But we found no difference on TMB of *TP53* and *ATR* mutations. No significant association between TMB and lymph node metastasis (*P* = 0.922), peritoneal metastasis (*P* = 0.220) and smoking status (*P* = 0.873) was found (data not shown).

## Discussion

Nowadays, targeted therapy has greatly improved the outcomes of patients with specific molecular changes. Therapeutic decisions should be guided by the molecular changes of tumor with standardized and easily interpretable annotation. Such detailed, evidence-based information about individual somatic mutations could be found on OncoKB (33, 34). In this study, we performed genomic analysis of 101 patients with LUAD in eastern China using a customized 639 genes DNA panel, which contributed to the selection of matched targeted therapies. Nearly 70% of patients harbored mutations that matched the targeted drugs, and 41.6% of patients with actionable alterations were recommended FDA-approved drugs. About 18.8% of patients had gene mutations recommended by NCCN guidelines or other professional guidelines. Almost 29.7% of the patients had no clinically applicable mutations of NSCLC, among which, 8% had mutations in *CEBPA* and *RB1*, and 6% had *TCF7L2* mutations, providing targets for clinical drug development for patients who did not have evidence of drugs. We were surprised to find that targeted therapy has become the first choice for patients with level_1 mutations. However, for patients with level_2 and below mutations, surgery and chemotherapy are still the first choice. Among the 71 patients with clinically actionable alterations, patients used matching targeted drugs, resulting in a better outcome, and 3 patients who had mutations of the recommended targeted drug, but they received chemotherapy and 1 patient without actionable alteration who was treated with chemotherapy showed worse prognosis than those received targeted agent, suggesting the significance of incorporation of NGS analysis into standard clinical practice. Though not all patients with mutations meet the indications for the use of targeted drugs, we consider and hope that patients with mutations who meet the indications can choose targeted drugs and benefit from them.

Furthermore, we found significant relationship between *CEBPA* mutations and the efficacy of *EGFR*-TKIs. Tumor heterogeneity is an important reason for the efficacy and resistance to EGFR targeted therapy in patients with NSCLC (35). Interestingly, we found that *CEBPA* mutations may be a biomarker for *EGFR*-TKIs, as the high frequency of *CEBPA* mutations indicated a poor prognosis of *EGFR*-TKIs treatment.

DNA panel can analyze the value of TMB which has become a predictive biomarker for ICI treatment. High TMB is known to be associated with DNA mismatch repair pathway genes and *TP53* (36, 37), but we did not detect a correlation of them. However, statistical analysis showed the significant association between TMB and mutations in *ERBB2*, *CEBPA*, *RB1* and *TCF7L2*, the top frequently mutated genes. If *EGFR*-TKI treatment is used, the efficacy of these patients may be poor, but patients may have higher TMB, which may point to the use of immunotherapeutic drugs, suggesting potential predictive biomarkers for response to PD-1 blockade immunotherapy.

The limitation of this study is that the sample size of patients with complete follow-up records is too small. In the future, more samples will be collected to elucidate the clinical value of targeted therapies based on NGS detection and to explore the clinical operability of using OncoKB database to guide patients’ treatment. Additionally, several novel mutated genes in the panel were found and they were closely associated with age, TNM stage and lymph node metastasis. Further study should be focused on the relationship between these mutations and clinical characteristics and signaling pathway to explore the mechanism of these genes leading to LUAD.

## Conclusion

In conclusion, genomic profile of 101 Chinese LUAD patients was identified. Novel genes with high mutation frequency such as *ERBB2*, *ATR*, *CEBPA*, *RB1* and *TCF7L2* were closely related to the efficacy of targeted therapies and TMB. NGS is conducive to guide patients to choose targeted drugs and provide basis for the development and application of precise treatment strategies for Chinese lung cancer patients.

## Data Availability Statement

The datasets presented in this study can be found in online repositories. The names of the repository/repositories and accession number(s) can be found below: NCBI-Sequence Read Archive (SRA), PRJNA774908.

## Ethics Statement

The studies involving human participants were reviewed and approved by the Ethics Committee of the Zhongshan Hospital Affiliated to Fudan University. The patients/participants provided their written informed consent to participate in this study.

## Author Contributions

JL and CL designed the manuscript. W-YX, MY, and ZL analyzed the data and wrote the manuscript. All the authors contributed to the article and approved the submitted version.

## Funding

Shanghai Municipal Key Clinical Specialty(shslczdzk02201), Shanghai Top-Priority Clinical Key Disciplines Construction Project (2017ZZ02013), Young Medical Professionals Training Program from Shanghai Medical and Health Development Foundation (Q2017-056).

## Conflict of Interest

W-YX was employed by company Singlera Genomics (Shanghai) Ltd.

The remaining authors declare that the research was conducted in the absence of any commercial or financial relationships that could be construed as a potential conflict of interest.

## Publisher’s Note

All claims expressed in this article are solely those of the authors and do not necessarily represent those of their affiliated organizations, or those of the publisher, the editors and the reviewers. Any product that may be evaluated in this article, or claim that may be made by its manufacturer, is not guaranteed or endorsed by the publisher.
