# Supplementary material for: Genetic Alteration Profiling of Chinese Lung Adenocarcinoma and Its Effect on Targeted Therapy Efficacy
Source: Front Oncol. 2021 Dec 14;11:726547. doi: 10.3389/fonc.2021.726547 (PMC8712938; doi:10.3389/fonc.2021.726547)

Supplementary Table 1. Genes list of the DNA panel

| ABCB1 | BRD4 | COL5A1 | EPCAM | FGFR3 | HIST3H3 | KMT2B | MSI2 | PCNA | PTPRT | SETD8 | TEK | ZFHX3 |
| --- | --- | --- | --- | --- | --- | --- | --- | --- | --- | --- | --- | --- |
| ABCB9 | BRIP1 | CREBBP | EPHA3 | FGFR4 | HLA-A | KMT2C | MST1 | PDCD1 | QKI | SF3B1 | TERT | ZNF217 |
| ABL1 | BTG1 | CRKL | EPHA5 | FH | HLA-B | KMT2D | MST1R | PDCD1LG2 | RAB35 | SGK1 | TET1 | ZNF703 |
| ABL2 | BTG2 | CRLF2 | EPHA7 | FLCN | HLA-C | KNSTRN | MTAP | PDGFRA | RAC1 | SH2B3 | TET2 |  |
| ACE2 | BTK | CSDE1 | EPHB1 | FLT1 | HLA-DRB1 | KRAS | MTHFR | PDGFRB | RAC2 | SH2D1A | TGFBR1 |  |
| ACVR1 | C10orf54 | CSF1R | EPHB4 | FLT3 | HMGB1 | LATS1 | MTOR | PDIA3 | RAD21 | SHOC2 | TGFBR2 |  |
| ACVR1B | C11orf30 | CSF3R | EPHX1 | FLT4 | HMGN1 | LATS2 | MTRR | PDK1 | RAD50 | SHQ1 | TIPARP |  |
| AGO2 | C8orf34 | CTCF | ERAP1 | FOXA1 | HNF1A | LGALS9 | MUTYH | PDPK1 | RAD51 | SLC34A2 | TMEM127 |  |
| AKT1 | CALR | CTLA4 | ERAP2 | FOXL2 | HOXB13 | LGMN | MYB | PGR | RAD51B | SLCO1B1 | TMPRSS2 |  |
| AKT2 | CANX | CTNNA1 | ERBB2 | FOXO1 | HRAS | LIG1 | MYC | PHF6 | RAD51C | SLIT2 | TNF |  |
| AKT3 | CARD11 | CTNNB1 | ERBB3 | FOXP1 | HSD3B1 | LIG3 | MYCL | PHOX2B | RAD51D | SLX4 | TNFAIP3 |  |
| ALK | CARM1 | CTSB | ERBB4 | FRS2 | HSP90AA1 | LMO1 | MYCN | PIK3C2B | RAD52 | SMAD2 | TNFRSF14 |  |
| ALOX12B | CASP8 | CTSL | ERCC1 | FUBP1 | ICOSLG | LNPEP | MYD88 | PIK3C2G | RAD54L | SMAD3 | TNFRSF9 |  |
| AMER1 | CBFB | CTSS | ERCC2 | FYN | ID3 | LRP1B | MYOD1 | PIK3C3 | RAF1 | SMAD4 | TNFSF14 |  |
| ANKRD11 | CBL | CUL3 | ERCC3 | GABRA6 | IDE | LTK | NBN | PIK3CA | RANBP2 | SMARCA4 | TNFSF18 |  |
| APC | CBR3 | CUL4A | ERCC4 | GATA1 | IDH1 | LYN | NCOA3 | PIK3CB | RARA | SMARCB1 | TNFSF4 |  |
| AR | CCND1 | CXCR4 | ERCC5 | GATA2 | IDH2 | LZTR1 | NCOR1 | PIK3CD | RASA1 | SMARCD1 | TNFSF9 |  |
| ARAF | CCND2 | CYLD | ERF | GATA3 | IFI30 | MAF | NEGR1 | PIK3CG | RB1 | SMO | TOP1 |  |
| ARFRP1 | CCND3 | CYP17A1 | ERG | GATA4 | IFNGR1 | MAGI2 | NF1 | PIK3R1 | RBM10 | SMYD3 | TOP2A |  |
| ARID1A | CCNE1 | CYP19A1 | ERRFI1 | GATA6 | IGF1 | MALT1 | NF2 | PIK3R2 | RECQL | SNCAIP | TP53 |  |
| ARID1B | CD200 | CYP2C19 | ESR1 | GID4 | IGF1R | MAP2K1 | NFE2L2 | PIK3R3 | RECQL4 | SOCS1 | TP53BP1 |  |
| ARID2 | CD22 | CYP2C8 | ETV1 | GLI1 | IGF2 | MAP2K2 | NFKBIA | PIM1 | REL | SOD2 | TP63 |  |
| ARID5B | CD274 | CYP2C9 | ETV4 | GNA11 | IKBKE | MAP2K4 | NKX2-1 | PLCG2 | RET | SOS1 | TP73 |  |
| ASXL1 | CD276 | CYP2D6 | ETV5 | GNA13 | IKZF1 | MAP3K1 | NKX3-1 | PLK2 | RFWD2 | SOX10 | TPMT |  |
| ASXL2 | CD40 | CYP3A4 | ETV6 | GNAQ | IL10 | MAP3K13 | NOTCH1 | PMAIP1 | RHEB | SOX17 | TPP2 |  |
| ATM | CD40LG | CYSLTR2 | EWSR1 | GNAS | IL7R | MAP3K14 | NOTCH2 | PMS1 | RHOA | SOX2 | TRAF2 |  |
| ATR | CD48 | DAXX | EXO1 | GPR124 | INHA | MAPK1 | NOTCH3 | PMS2 | RICTOR | SOX9 | TRAF7 |  |
| ATRX | CD70 | DCUN1D1 | EZH1 | GPS2 | INHBA | MAPK3 | NOTCH4 | PNRC1 | RIT1 | SPEN | TSC1 |  |
| AURKA | CD74 | DDR1 | EZH2 | GREM1 | INPP4A | MAPKAP1 | NPEPPS | POLB | RNF43 | SPOP | TSC2 |  |
| AURKB | CD79A | DDR2 | EZR | GRIN2A | INPP4B | MAX | NPM1 | POLD1 | ROS1 | SPRED1 | TSHR |  |
| AXIN1 | CD79B | DHFR | FAM175A | GRM3 | INPPL1 | MCL1 | NQO1 | POLE | RPS6KA4 | SPTA1 | TYMS |  |
| AXIN2 | CD80 | DICER1 | FAM46C | GSK3B | INSR | MDC1 | NRAS | PPARG | RPS6KB2 | SRC | TYRO3 |  |
| AXL | CD86 | DIS3 | FAM58A | GSTP1 | IRF2 | MDM2 | NRD1 | PPM1D | RPTOR | SRSF2 | U2AF1 |  |
| B2M | CDA | DMD | FANCA | H3F3A | IRF4 | MDM4 | NSD1 | PPP2R1A | RRAGC | STAG2 | UGT1A1 |  |
| BABAM1 | CDC42 | DNAJB1 | FANCC | H3F3B | IRS1 | MED12 | NT5C2 | PPP2R2A | RRAS | STAT3 | UGT1A9 |  |
| BAP1 | CDC73 | DNMT1 | FANCD2 | H3F3C | IRS2 | MEF2B | NTHL1 | PPP4R2 | RRAS2 | STAT4 | UPF1 |  |
| BARD1 | CDH1 | DNMT3A | FANCE | HDAC1 | ITGAV | MEN1 | NTRK1 | PPP6C | RRM1 | STAT5A | VEGFA |  |
| BBC3 | CDK12 | DNMT3B | FANCF | HERC1 | ITGB3 | MERTK | NTRK2 | PRDM1 | RSPO2 | STAT5B | VHL |  |
| BCL10 | CDK4 | DOT1L | FANCG | HGF | JAK1 | MET | NTRK3 | PRDM14 | RTEL1 | STK11 | VTCN1 |  |
| BCL2 | CDK6 | DPYD | FANCL | HIST1H1C | JAK2 | MGA | NUF2 | PREX2 | RUNX1 | STK19 | WHSC1 |  |
| BCL2L1 | CDK8 | DROSHA | FAS | HIST1H2BD | JAK3 | MICA | NUP93 | PRKAR1A | RUNX1T1 | STK40 | WHSC1L1 |  |
| BCL2L11 | CDKN1A | DUSP4 | FAT1 | HIST1H3A | JUN | MICB | NUTM1 | PRKCI | RXRA | SUFU | WISP3 |  |
| BCL2L2 | CDKN1B | DYNC2H1 | FBXW7 | HIST1H3B | KAT6A | MITF | P2RY8 | PRKD1 | RYBP | SUZ12 | WT1 |  |
| BCL6 | CDKN2A | E2F3 | FGF10 | HIST1H3C | KDM5A | MKNK1 | PAK1 | PRKDC | SDC4 | SYK | WWTR1 |  |
| BCOR | CDKN2B | EED | FGF12 | HIST1H3D | KDM5C | MLH1 | PAK3 | PRSS8 | SDHA | TAF1 | XIAP |  |
| BCORL1 | CDKN2C | EGFL7 | FGF14 | HIST1H3E | KDM6A | MLH3 | PAK7 | PTCH1 | SDHAF2 | TAP1 | XPC |  |
| BCR | CEBPA | EGFR | FGF19 | HIST1H3F | KDR | MPL | PALB2 | PTEN | SDHB | TAP2 | XPO1 |  |
| BIRC3 | CENPA | EIF1AX | FGF23 | HIST1H3G | KEAP1 | MRE11 | PARK2 | PTGS2 | SDHC | TAPBP | XRCC1 |  |
| BLM | CHD2 | EIF4A2 | FGF3 | HIST1H3H | KEL | MRE11A | PARP1 | PTP4A1 | SDHD | TAPBPL | XRCC2 |  |
| BMPR1A | CHD4 | EIF4E | FGF4 | HIST1H3I | KIT | MSH2 | PARP2 | PTPN11 | SESN1 | TBX3 | XRCC5 |  |
| BRAF | CHEK1 | ELF3 | FGF6 | HIST1H3J | KLF4 | MSH3 | PARP3 | PTPRD | SESN2 | TCEB1 | YAP1 |  |
| BRCA1 | CHEK2 | EP300 | FGFR1 | HIST2H3C | KLHL6 | MSH6 | PAX5 | PTPRO | SESN3 | TCF3 | YES1 |  |
| BRCA2 | CIC | EPAS1 | FGFR2 | HIST2H3D | KMT2A | MFAT31 | PBRM1 | PTPRS | SETD2 | TCF7L2 | ZBTB2 |  |

The DNA panel is a hybridization capture-based NGS panel to detect single nucleotide variants (SNVs), insertion and deletion alterations (InDels) and copy number alterations (CNAs) involved in 639 cancer associated genes. This panel is designed to provide mutation profiling of patients with solid tumors.

Supplementary Table 2. Description of 101 Chinese LUAD cases

| Variable | Cases  N = 101 |
| --- | --- |
| **Age (years)** |  |
| Median | 61 |
| Range | 34-88 |
| **Gender, n (%)** |  |
| Male | 42 (41.6) |
| Female | 59 (58.4) |
| **TNM stage, n (%)** |  |
| I | 47 (46.5) |
| II | 17 (16.8) |
| III | 4 (4.0) |
| IV | 33 (32.7) |
| **Differentiation, n (%)** |  |
| Well  Moderate  Poor | 4 (4.0)  56 (55.4)  41 (40.6) |
| **Smoking status, n (%)** |  |
| Non-smokers | 82 (81.2) |
| Ever-smokers | 7 (6.9) |
| Current smokers | 12 (11.9) |
| **Lymph node metastasis, n (%)** |  |
| Negative | 64 (63.4) |
| Positive | 37 (36.6) |
| **Visceral pleura invasion, n (%)** |  |
| No | 60 (59.4) |
| Yes | 41 (40.6) |

Abbreviations: LUAD, lung adenocarcinoma.

Supplementary Table 3. Comparison of the frequency of selected genes between Chinese cohort and TCGA and ICGC cohorts

|  | *EGFR* | *P* | *TP53* | *P* | *ERBB2* | *P* | *ATR* | *P* | *CEBPA* | *P* | *RB1* | *P* | *TCF7L2* | *P* | *ROS1* | *P* | *SPTA1* | *P* |
| --- | --- | --- | --- | --- | --- | --- | --- | --- | --- | --- | --- | --- | --- | --- | --- | --- | --- | --- |
| Chinese cohort | 53 |  | 32 |  | 25 |  | 20 |  | 16 |  | 16 |  | 14 |  | 12 |  | 12 |  |
| Asian cohort (TCGA) | 46.5 | 0.358 | 36.5 | 0.503 | 4.2 | <0.0001 | 2.6 | 0.0001 | 0.1 | <0.0001 | 3.5 | 0.003 | 0.3 | 0.0002 | 2.2 | 0.007 | 10.3 | 0.703 |
| European cohort (TCGA) | 16 | <0.0001 | 52.7 | 0.003 | 2.4 | <0.0001 | 4.7 | 0.001 | 0.1 | <0.0001 | 3.6 | 0.003 | 2.4 | 0.003 | 4.1 | 0.04 | 31.4 | 0.001 |
| American cohort (ICGC) | 10.27 | 0 | 48.26 | 0.019 | 3.29 | <0.0001 | 4.07 | 0.001 | 0.1 | <0.0001 | 5.43 | 0.016 | 1.94 | 0.002 | 5.43 | 0.1 | 32.36 | 0.001 |
|  | *NCOR1* | *P* | *PRKD1* | *P* | *AXIN1* | *P* | *CARD11* | *P* | *FGFR4* | *P* | *MLH1* | *P* | *MSH3* | *P* | *POLD1* | *P* | *STAG2* | *P* |
| Chinese cohort | 10 |  | 10 |  | 9 |  | 9 |  | 9 |  | 9 |  | 9 |  | 9 |  | 9 |  |
| Asian cohort (TCGA) | 1.9 | 0.15 | 0.6 | 0.003 | 0.3 | 0.003 | 1 | 0.009 | 1 | 0.009 | 0.6 | 0.005 | 0.6 | 0.005 | 0 | 0.002 | 1 | 0.009 |
| European cohort (TCGA) | 4.7 | 0.151 | 1.8 | 0.014 | 1.2 | 0.012 | 5.3 | 0.31 | 0.6 | 0.005 | 2.4 | 0.044 | 1.8 | 0.024 | 2.4 | 0.044 | 5.9 | 0.404 |
| American cohort (ICGC) | 5.23 | 0.204 | 5.23 | 0.204 | 3.49 | 0.107 | 7.75 | 0.75 | 3.49 | 0.107 | 4.07 | 0.158 | 1.94 | 0.028 | 2.13 | 0.034 | 11.05 | 0.629 |

*P*- value represents the statistically significant difference between the mutation frequency of Chinese population detected by this DNA panel and that reported in TCGA database or ICGC database.


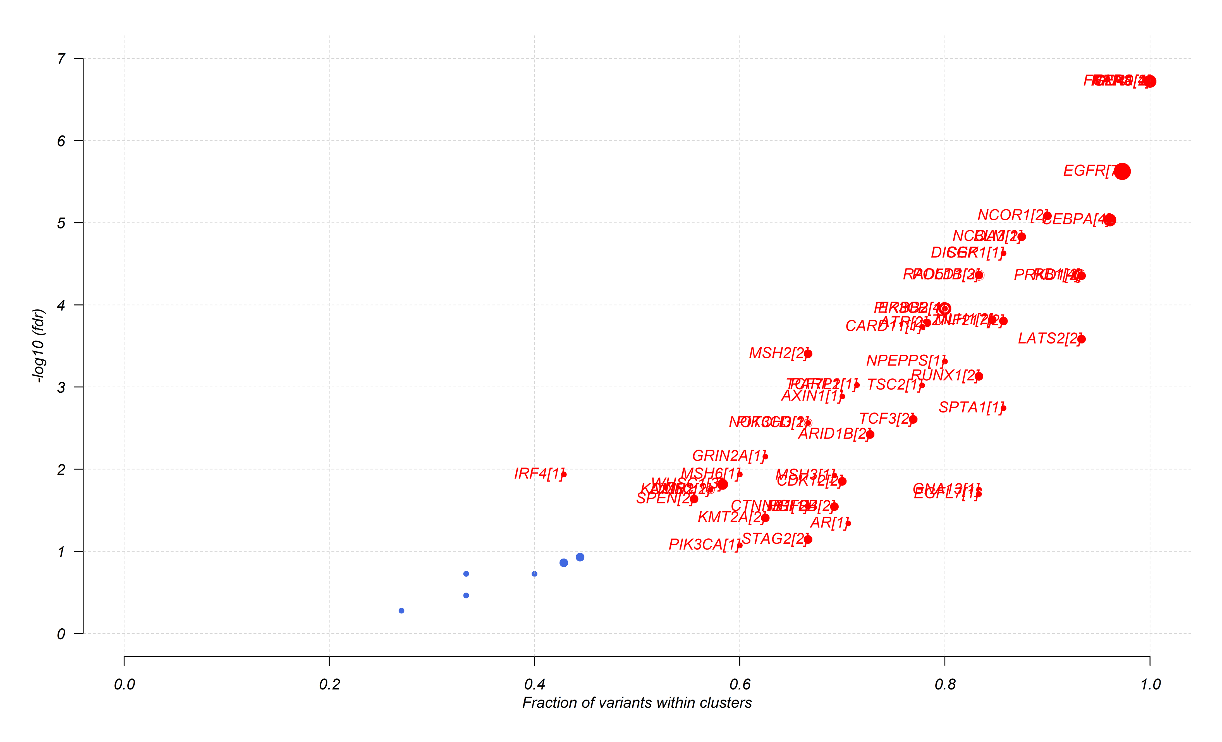


Supplementary Figure 1. Detecting cancer driver genes via algorithm. The size of the dots shows the number of clusters in the gene.


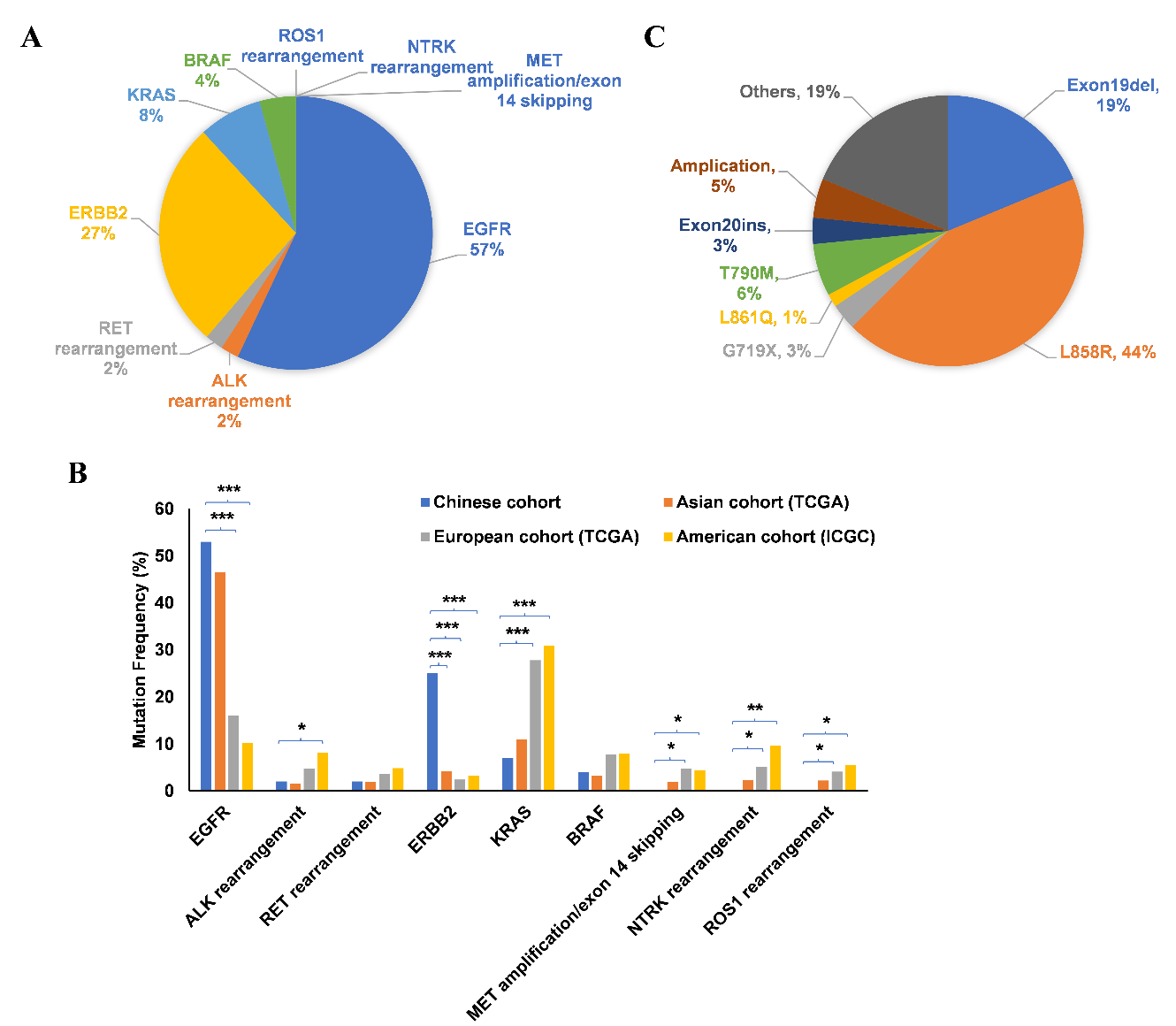


Supplementary Figure 2. The spectrum of driver gene mutations. (A) Mutation frequency of driver genes in the current Chinese cohort. (B) Comparison of driver gene mutation frequency among the four groups. (C) Distribution of *EGFR* mutation subtypes in treatment-native patients. ****p* < 0.001, ***p* < 0.01 and **p* < 0.05.


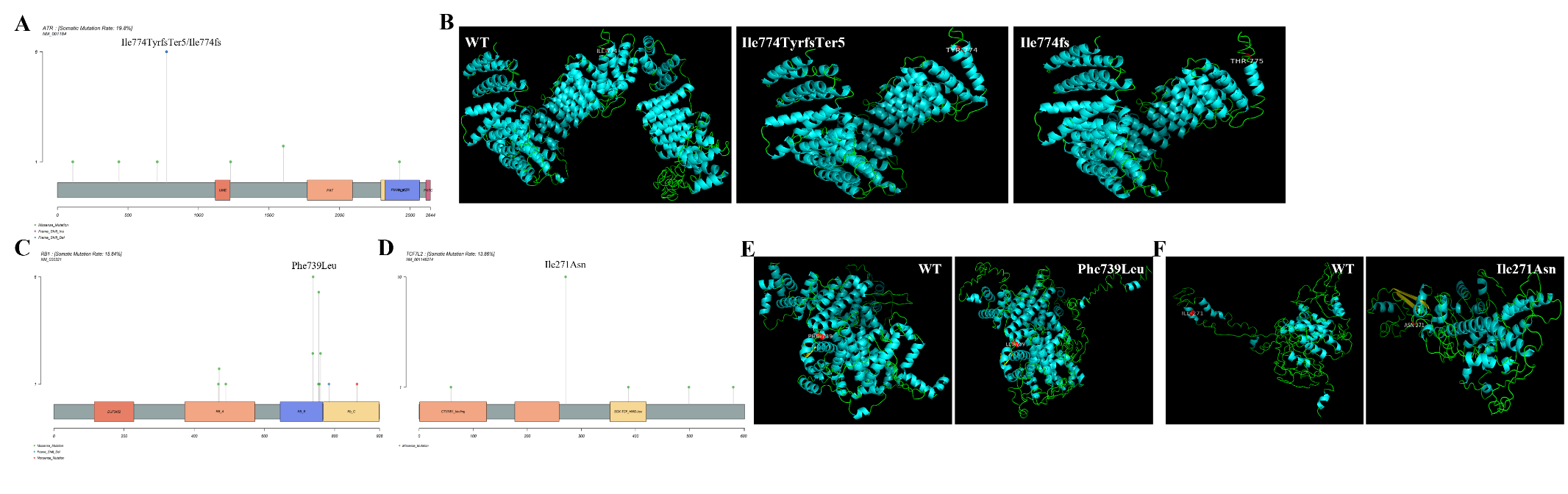


Supplementary Figure 3. Mutant points and 3-D protein structures of normal and mutant protein of ATR (A, B), RB1 and TCF7L2 (C-F). Blue means helix, green means sheet. The mutant points were shown in red in the structure diagram.

Supplementary Figure 4. The mutated sites of genes with high mutation frequency


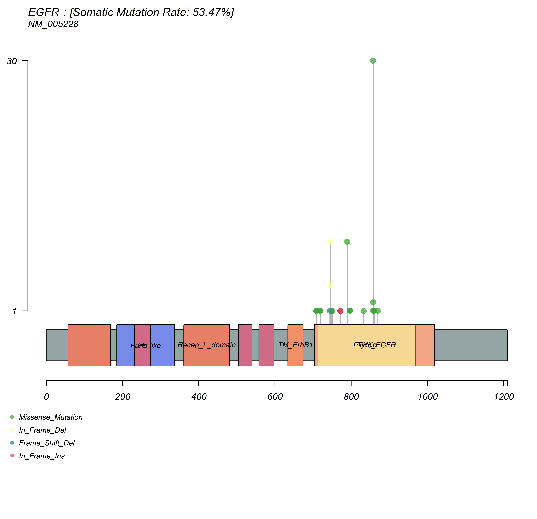

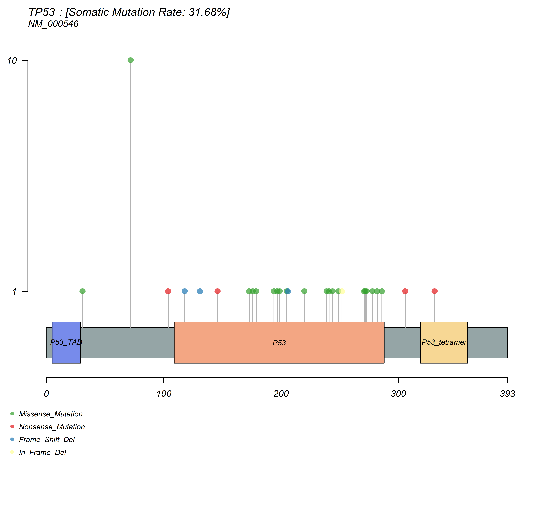

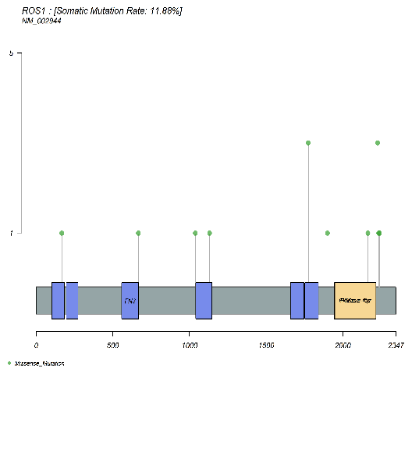

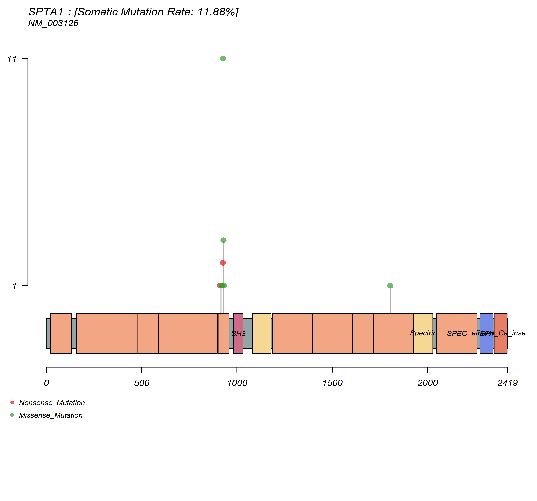

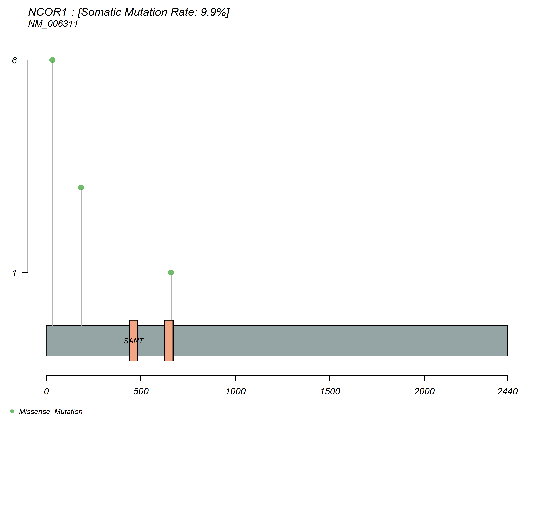

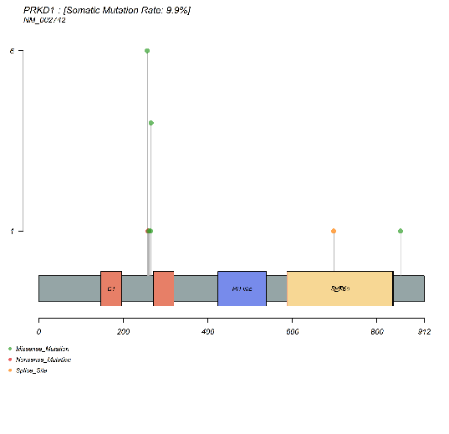

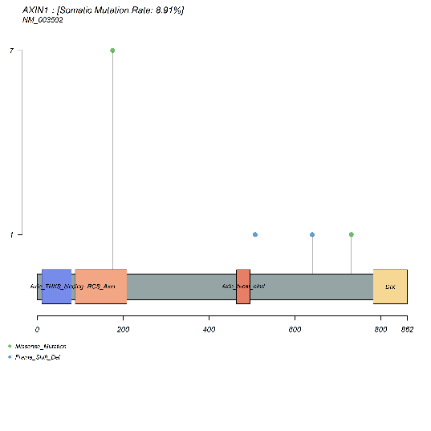

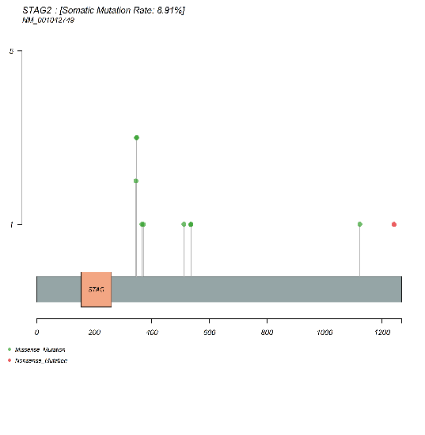

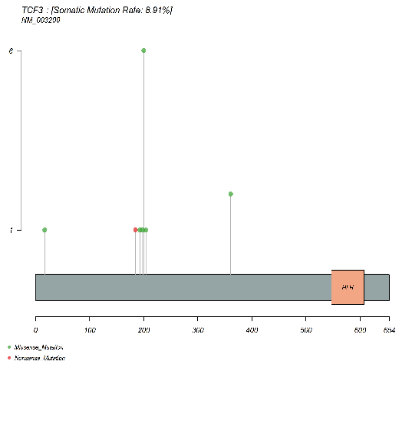

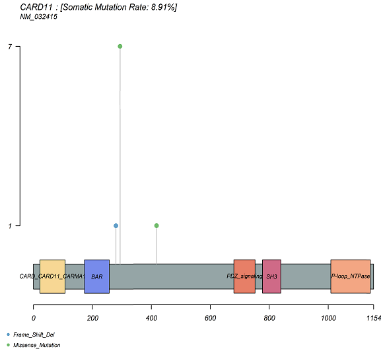

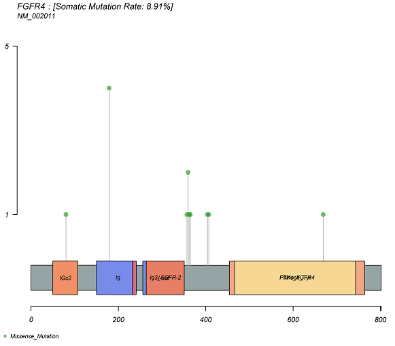

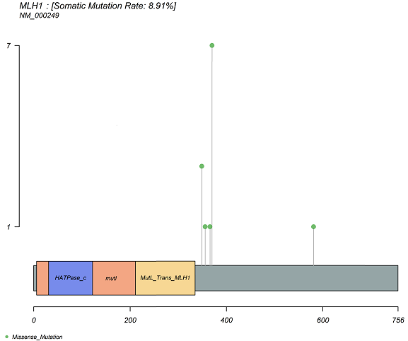

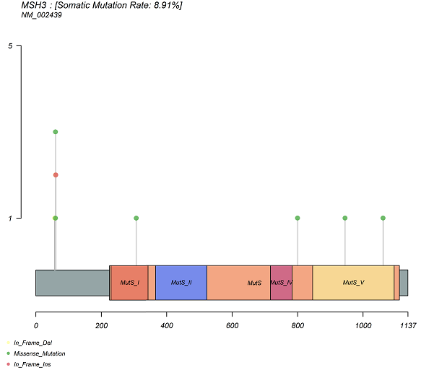

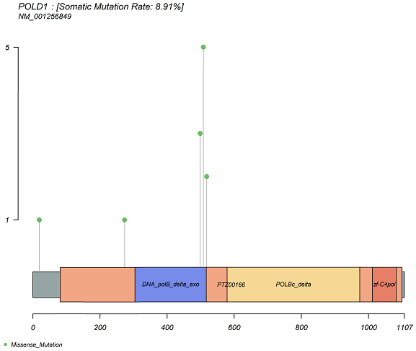

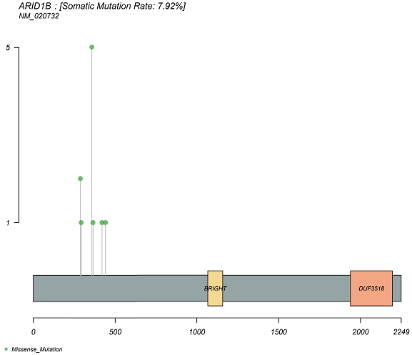

Supplement: Supplementary file 1 [file DataSheet_1.docx]
